# Quality of life in patients with breast cancer and their rehabilitation needs

**DOI:** 10.12669/pjms.301.3952

**Published:** 2014

**Authors:** Chen Hong-li, Wang Xiao-chun, Wang Jiang-bin, Zhang Jing-bo, Wang Yao

**Affiliations:** 1 Chen Hong-li, Nursing Department, The First Affiliated Hospital of Harerbin Medical University, Harerbin, China.; 2 Wang Xiao-chun, Nursing Department, The First Affiliated Hospital of Harerbin Medical University, Harerbin, China.; 3 Wang Jiang-bin, Nursing Department, The First Affiliated Hospital of Harerbin Medical University, Harerbin, China.; 4 Zhang Jing-bo, The Stomach and Spleen Portal Hypertension Surgery, The First Affiliated Hospital of Harerbin Medical University, Harerbin, China.; 5 Wang Yao, Nurse Practitioner, Experimental Surgery, The First Affiliated Hospital of Harerbin Medical University, Harerbin, China.

**Keywords:** Breast cancer, Quality of life, FACT-B, Semi-structured interview

## Abstract

***Objective: ***We conducted a cross sectional study to investigate the quality of life (QOL) in breast cancer patients after treatment for one year and identify factors which may facilitate improvements in health care for breast cancer.

***Methods:*** A total of 154 patients of breast cancer were collected from The First Affiliated Hospital of Harerbin Medical University during May 2008 and May 2010, and they were divided into three groups. The quality of life was assessed by Functional assessment of cancer therapy- breast (FACT-B) version 4, and a semi-structured interview was used to investigate the information and rehabilitation needs of the breast cancer patients.

***Results*** *: *Group II had the best social well-being, functional well-being and Total FACT-G among the three groups. Group III had the best physical well-being, emotional well-being, breast specific subscales, total FACT-B and TOI among the three groups. Higher PWB scores were significantly correlation with lower tumor stage; increased SWB scores were significantly correlated with education and occupation, and lower EWB scores were correlated with younger aged women and higher tumor stage (< 40 years). The semi-structured investigation showed all of them want to receive tumor markers detection and PET scan to prevent recurrence. 56% of these patients were worried about symptoms. 42% of the patients reported they had restriction in sexual relationship, and 57% wanted to improve their body image and reconstruction surgery.

***Conclusions:*** Breast cancer patients should be followed up for their quality of life and provided effective therapy for their physical and psychological problems.

## INTRODUCTION

Breast cancer is the most frequently diagnosed cancer in women, with an estimated 1.38 million new cancer cases diagnosed in 2008.^1^^,^^2^ Advances in diagnostic and trewatment could result in increased survival. Therefore, coping with breast as a chronic disease has become a common phenomenon.

The increased survival of breast cancer patients, the younger age at diagnosis, and the more focus should be taken on quality of life (QOL) to both health care providers and patients. Previous evidence has indicated that breast cancer patients may not show obvious evidence of disease, but they do suffer from a number of problems which persist long after initial treatment,^3^^-^^6^ such as physical problems (pain and fatigue), psychological problems (fear of recurrence and inability to cope with the disease), and psychosocial problems(family worries and sexual problems).^7^ Therefore, there is a great need to provide education, information and support over time.

 There are very few studies in Chinese patients concerning the quality of life in breast cancer patients.^8^^,^^9^ However, none of them have studied on the long term adaptation during the follow up. Hence we conducted a cross sectional study to investigate the QOL in breast cancer patients after treatment for one year and identify factors which may facilitate improvements in health care for breast cancer.

## METHODS

***Patients: ***This study is a descriptive cross sectional study. A total of 154 patients of breast cancer were collected from the Second Affiliated Hospital of Guangzhou Medical University during May 2008 and May 2010. Patients who were diagnosed with local or locoregional breast cancer and had undergone surgery between 2008 and 2010 and were followed up in the study. Patients with metastatic breast cancer or other systemic illnesses such as diabetes, depression, coronary artery disease were excluded.

All patients either underwent initial surgery followed by adjuvant chemotherapy or neoadjuvant chemotherapy followed by surgery. Written consent was obtained from all the patients. The study was approved by the Ethics committee of the Second Affiliated Hospital of Guangzhou Medical University.

***Study instruments: ***The QOL of breast cancer patients was assessed using Functional assessment of Cancer Therapy - Breast (FACT-B) version 4.^10^ This instrument has both a generic part (FACT-G) and a breast cancer specific module (BSS). This questionnaire has good validity and reliability properties.^11^^-^^12^

 It has a 36 item self administered scale consisting of 4 general subscales, including physical well being (PWB), social well being ( SWB), Functional well being (FWB), and emotional well being (EWB). The fifth subscale contains 9 items for breast cancer (BSS). The instrument has multiple scoring options: subscale scores, total score (FACT–B and FACT–G) and Trial outcome index (TOI). FACT –G has a stronger focus on social and emotional aspects. TOI which is the sum of PWB, FWB and BCS is an efficient summary of index of physical/ functional aspects. On the first week of enrolling into study, face-to-face interviews were conducted to evaluate the QOL of participants.

The follow-up of all patients were conducted every three months during first one year after initial treatment, semi-annually for the next two years after initial treatment. A semi-structured interview to identify measures which would help overcome restriction in life yielded some interesting findings. As the QOL issues may change during the follow-up period, we divided all patients into three groups according to the duration of follow up (Group 1: one year; Group II: two year; and Group III: five years).

Patients’ information was collected from their medical records. Sociodemographic variables included age at diagnosis, marital status, level of education and occupation. Clinical variables included tumor stage, types of surgery, status, hormonal therapy, chemotherapy and radiotherapy as well as recurrence.

***Statistical analysis*** *: *The SPSS version 13.0 software (SPSS Inc, Chicago, IL) was used for statistical analysis. A two-sided P value <0.05 was determined as statistically significant. Continuous variables were expressed as mean ± standard deviation (SD), while categorical variables were shown as frequencies and percentages. One way ANOVA was conducted for comparison of socio demographic and clinical data among the three groups. FACT-B subscales were compared among the three groups by Covariance (ANCOVA) after adjusting for variables for age, religion, marital status, chemotherapy and radiotherapy. Spearman correlation analysis was taken to evaluate the association of demographic and clinical variables with FACT-B subscale scores.

## RESULTS

A total of 154 patients participated into our study and completed the questionnaires. There were 64 patients in group I, 48 in group II and 42 in group III. The demographic and clinical characteristics of included population are shown in Table-I. The mean ages of group I, II and III were 47.4 years, 43.3 years and 59.1 years, respectively.

Majority of patients were at clinical stage II and III at the time of diagnosis, with a proportion of 49.4% and 30.5%, respectively. Modified radical mastectomy was the most commonly performed procedure (92.2%), and almost all patients received chemotherapy and hormone therapy. Majority of the patients (80%) did not show local or systemic recurrence during the follow-up period. 

The QOL subscales in the three groups are summarized in Table-II. Group II had the best social well-being, functional well-being and Total FACT-G among the three groups. Group III had the best physical well-being, emotional well-being, breast specific subscales, total FACT-B and TOI among the three groups.

Association between demographic and clinical variables and QOL was evaluated by spearman correlation analysis (Data not shown). Higher PWB scores were significantly correlation with lower tumor stage (r=0.81, p=0.013); increased SWB scores were significantly correlated with education and occupation (r=0.88, p=0.003; r=0.76p=0.028), and lower EWB scores were correlated with younger aged women and higher tumor stage (< 40 years) (r=0.69, p= 0.034; r=0.84, p=0.01). . Moreover, lower BSS scores were correlated with lower tumor stage, chemotherapy and radiotherapy. 

We used semi-structured interview to investigate the information and rehabilitation needs of the breast cancer patients (Table-III). All the patients wanted to have more information about their disease, such as treatment measures, life expectancy, and expense for the further treatment and health care. All of them want to receive tumor markers detection and PET scan to prevent recurrence. 56% of these patients were worried about symptoms, including pain in arm and shoulder, lack of energy, limb swelling and effective remedy. 42% of the patients reported they had restriction in sexual relationship, and 57% wanted to improve their body image and reconstruction surgery. 35% of the patients had felt difficulty in further medical treatment mainly due to economy problems.

## DISCUSSION

This is perhaps the first study in China to assess quality of life of breast cancer patients and their requirement for further medical treatment. Previous evidence indicated that breast cancer survivors suffer from various physical and psychological problems, and they want to get rehabilitation and medical care services.^13^ Identification of these problems could help clinician and healthcare planners to take related measures for these survivors and make policy to provide more services to them. 

**Table-I:** Demographic and clinical characteristics of study groups

*Variables*		*Group I*	*Group II*	*Group III*
		*N=64*	*N=48*	*N=42*
Mean age (years)		47.4±8.8	43.3±10.3	59.1±9.37
Marital Status	Single	0	0	0
	Married	64 (100%)	45 (93.8%)	32 (76.22%)
	Widowed	0	3 (10.4%)	10 (23%)
Education	Illiterate	41 (64.1%)	29 (60.4%)	32 (76%)
	Can read & write	23 (35.9%)	19 (39.6%)	10 (24%)
Occupation	Unemployed	60 (93.8%)	42 (87.5%)	39 (93%)
	Employed	4 (6.2%)	6 (12.5%)	3 (17%)
Tumor stage	I	21 (32.8%)	8 (16.6%)	2 (4.8%)
	II	27 (42.2%)	24 (50%)	25 (59.5%)
	III	16 (25.0%)	16(33.3%)	15 (35.7%)
Surgery	MRM	57 (89.1%)	43 (89.5%)	42 (100%)
	BCT	5 (7.8%)	5 (10.4%)	0
	MRM with Reconstruction	2 (3.1%)	0	0
Chemotherapy	Yes	61(95.3%)	44(91.7%)	39(92.9%)
	No	3(4.7%)	4(8.3%)	3(7.1%)
Radiotherapy	Yes	20 (31.2%)	10 (21%)	4 (9.5%)
	No	44 (68.8%)	38 (79%)	38 (90.5%)
Hormonal therapy	Yes	62 (96.9%)	46 (96%)	42 (100%)
	No	2 (3.1%)	2 (4%)	0
Recurrence	No recurrence	57 (89%)	36 (75%)	30 (71.3%)
	Local recurrence	3 (4.7%)	5 (10.4%)	9(21.5%)
	Systemic recurrence	4 (6.3%)	7 (14.5%)	3 (7.2%)

**Table-II:** QOL of three groups

*FACT-B Subscales*	*Group I*	*Group II*	*Group III*
	*(mean±SD)*	*(mean±SD)*	*(mean±SD)*
Physical well being (PWB)	17.06±5.30	17.00±7.66	17.33±4.05*
Social well being (SWB)	17.59±3.92	19.40±5.08*	17.28±2.70
Emotional well being (EWB)	14.84±4.52	16.41±5.39	16.58±3.26*
Functional well being (FWB)	13.28±4.08	14.41±6.10*	13.62±4.42
Breast specific subscales (BSS)	20.28±3.37	18.53±2.94	21.67±3.77*
Total FACT-B	81.06±14.60	85.75±20.15	88.83±12.80*
Total FACT-G	60.78±13.27	67.22±18.96*	66.87±10.76
TOI	48.63±9.50	49.94±14.51	55.67±9.98*

*Highest score on each scale

**Table-III:** Information and rehabilitation needs of the breast cancer patients

*Indexes*	*Answer*	*Patients, n (%)*
Know more about their disease	Yes	100%
	No	-
Receive tumor markers detection and PET scan	Yes	100%
	No	-
Have physical symptoms	Yes	56%
	No	44%
Have restriction in sexual relationship	Yes	42%
	No	58%
Want to improve upon their body image	Yes	57%
	No	46%
Have difficulty in further treatment	Yes	35%
	No	65%

Our study indicated that the total FACT-B score in Chinese breast cancer survivors are relatively lower than that in western cases. Previous study reported that the FACT-B score in American breast cancer cases were as high as 110.82 ± 19.0, which is significantly higher than that in our study.^14^ The lower score might be due to the lower education level, unemployment, low income and poor healthcare facility when compared with western countries. 

From our study, we found the breast cancer patients could cope well with the stress of treatment of breast cancer and bounce back to normal life after treatment for one year, such as a higher PWB, SWB and FWB in group I. However, the BSS, total FACT-B and TOI were significantly increased after treatment for five years. Patients in the first year of treatment are well recovered from physical, social and functional domains, but the emotion and breast specific domains need time to recovery. Our study is in line with previous studies conducted in American, Australian and Korean populations.^15^^-^^18^ Ganz et al reported that maximum physical and psychological recovery was achieved 1 year after initial treatment. ^18^

During the second follow-up year, our study finds the QOL were significantly improved in domains of SWB, FWB and total FACT-G, while the scores of these domains were decreased after treatment for five years. This finding suggests that the recreational activities, body image, sexual interest and sexual functioning of the breast cancer survivors may be deteriorated. A previous study had indicated that various problems which persisted long after initial treatment.^3^

Our study finds that all the cancer patients wanted to get more knowledge about their disease and receive tumor markers detection and PET scan, 42% of the patients have restriction in sexual relationship and 57% of them have body image, which indicated that many breast cancer survivors have problems in social and functional and physical domains. Previous studies have reported a high rate of sexuality concerns in breast cancer survivors.^19^^-^^22^ The reasons might be lack of physical attractiveness after breast surgery and other somatic problems such as vaginal dryness caused by chemotherapy.

In conclusion, there is a need to provide education, support and medical care to breast cancer patients. Breast cancer patients should be followed up for their QOL and provide effective therapy for their physical and psychological problems. It is warranted to establish health care system to follow up these breast cancer patients so as to help them overcome physical and psychological problems.
